# Supplementary material for: Insights of Expression Profile of Chemokine Family in Inflammatory Bowel Diseases and Carcinogenesis
Source: Int J Mol Sci. 2024 Oct 9;25(19):10857. doi: 10.3390/ijms251910857 (PMC11476924; doi:10.3390/ijms251910857)
Supplement: Supplementary file 1 [file ijms-25-10857-s001.zip › Supplemental table. Gene and probes in this study.pdf]

**Supplemental Table S1. Gene and probes in this study**

| Gene | Probe | Gene | Probe |
|---|---|---|---|
| CXCL1 | 207094_at | CXCR1 | 207094_at |
| CXCL2 | 209774_x_at | CXCR2 | 207008_at |
| CXCL3 | 207850_at | CXCR3 | 207681_at |
| CXCL4 | 206390_x_at | CXCR4 | 217028_at |
| CXCL5 | 214974_x_at | CXCR5 | 216734_s_at |
| CXCL6 | 206336_at | CXCR6 | 206974_at |
| CXCL7 | 214146_s_at | CXCR7 | 212977_at |
| CXCL8 | 202859_x_at | CCR1 | 205098_at |
| CXCL9 | 203915_at | CCR2 | 206978_at |
| CXCL11 | 210163_at | CCR3 | 208304_at |
| CXCL12 | 209687_at | CCR4 | 208376_at |
| CXCL13 | 205242_at | CCR5 | 206991_s_at |
| CXCL14 | 218002_s_at | CCR6 | 206983_at |
| CCL1 | 207533_at | CCR7 | 206337_at |
| CCL2 | 216598_s_at | CCR8 | 208059_at |
| CCL3 | 205114_s_at | CCR9 | 207445_s_at |
| CCL4 | 204103_at | CCR10 | 220565_at |
| CCL5 | 1405_i_at | CX3CR1 | 205898_at |
| CCL7 | 208075_s_at | XCR1 | 221468_at |
| CCL8 | 214038_at | | |
| CCL11 | 210133_at | | |
| CCL14 | 206407_s_at | | |
| CCL15 | 210390_s_at | | |
| CCL16 | 207354_at | | |
| CCL17 | 207900_at | | |
| CCL18 | 32128_at | | |
| CCL19 | 210072_at | | |
| CCL20 | 205476_at | | |
| CCL21 | 204606_at | | |
| CCL22 | 207861_at | | |
| CCL23 | 210548_at | | |
| CCL24 | 221463_at | | |
| CCL25 | 206988_at | | |
| CCL27 | 207955_at | | |
| CX3CL1 | 203687_at | | |
| XCL1 | 206365_at | | |
| XCL2 | 214567_s_at | | |

**Supplemental Table S2. Homologous relationship between mouse and human genes**

| **Human orthologous gene** | **Mouse orthologous gene** |
|---|---|
| CXCL1， CXCL2， CXCL3 | CXCL1， CXCL2， CXCL3 |
| CXCL4 | CXCL4 |
| CXCL5,6 | CXCL5 |
| CXCL7 | CXCL7 |
| CXCL8 | / |
| CXCL9 | CXCL9 |
| CXCL10 | CXCL10 |
| CXCL11 | CXCL11 |
| CXCL12 | CXCL12 |
| CXCL13 | CXCL13 |
| CXCL14 | CXCL14 |
| / | CXCL15 |
| CXCL16 | CXCL16 |
| CXCL17 | CXCL17 |
| CCL1 | CCL1 |
| CCL2,13 | CCL2,12 |
| CCL3, CCL18, CCL3L3 | CCL3 |
| CCL4 | CCL4 |
| CCL5 | CCL5 |
| CCL7 | CCL7 |
| CCL8 | CCL8 |
| CCL11 | CCL11 |
| CCL14 | / |
| CCL15, 23 | CCL6, 9 |
| CCL16 | / |
| CCL17 | CCL17 |
| CCL19 | CCL19 |
| CCL20 | CCL20 |
| CCL21 | CCL21a,b,d |
| CCL22 | CCL22 |
| CCL24 | CCL24 |
| CCL25 | CCL25 |
| CCL26 | CCL26 |
| CCL27 | CCL27a,b |
| CCL28 | CCL28 |
| XCL1, XCL2 | XCL1 |
| CX3CL1 | CX3CL1 |
