# Supplementary material for: Insights of Expression Profile of Chemokine Family in Inflammatory Bowel Diseases and Carcinogenesis
Source: Int J Mol Sci. 2024 Oct 9;25(19):10857. doi: 10.3390/ijms251910857 (PMC11476924; doi:10.3390/ijms251910857)
Supplement: Supplementary file 1 [file ijms-25-10857-s001.zip › Supplmental Figure.pptx]

## Slide 1
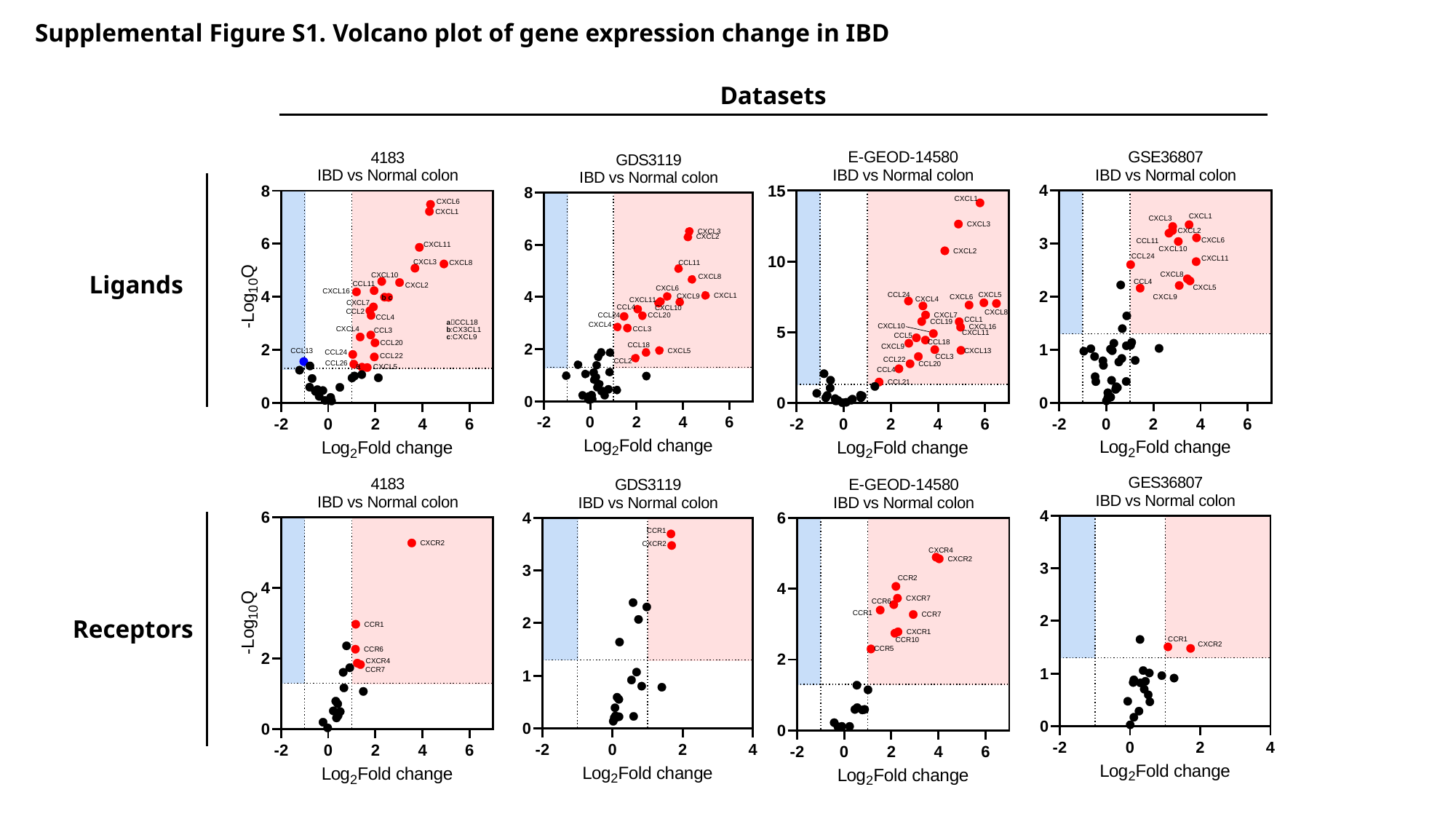

# Supplemental Figure S1. Volcano plot of gene expression change in IBD
Datasets
Ligands
Receptors

## Slide 2
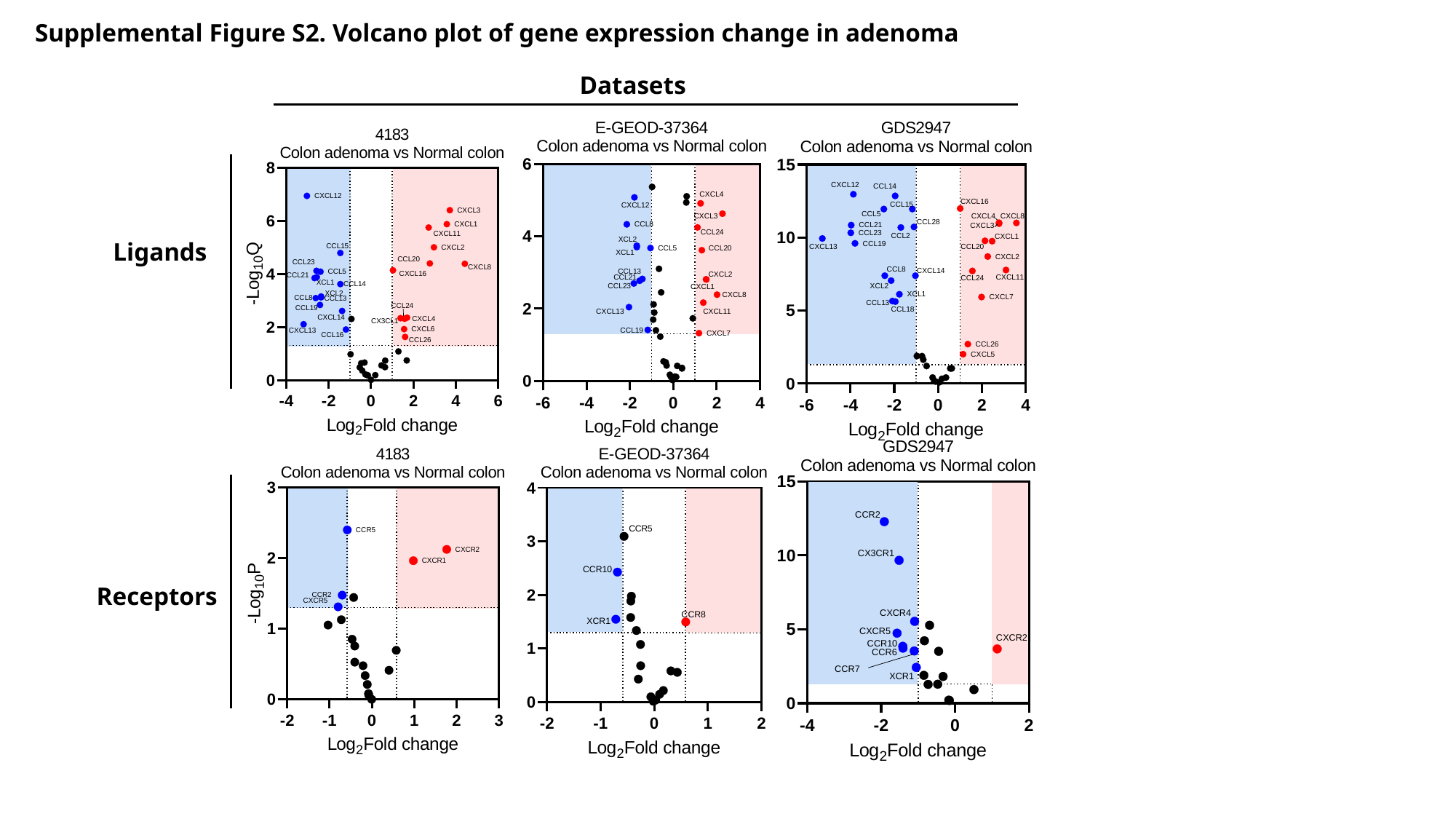

# Supplemental Figure S2. Volcano plot of gene expression change in adenoma
Datasets
Ligands
Receptors

## Slide 3
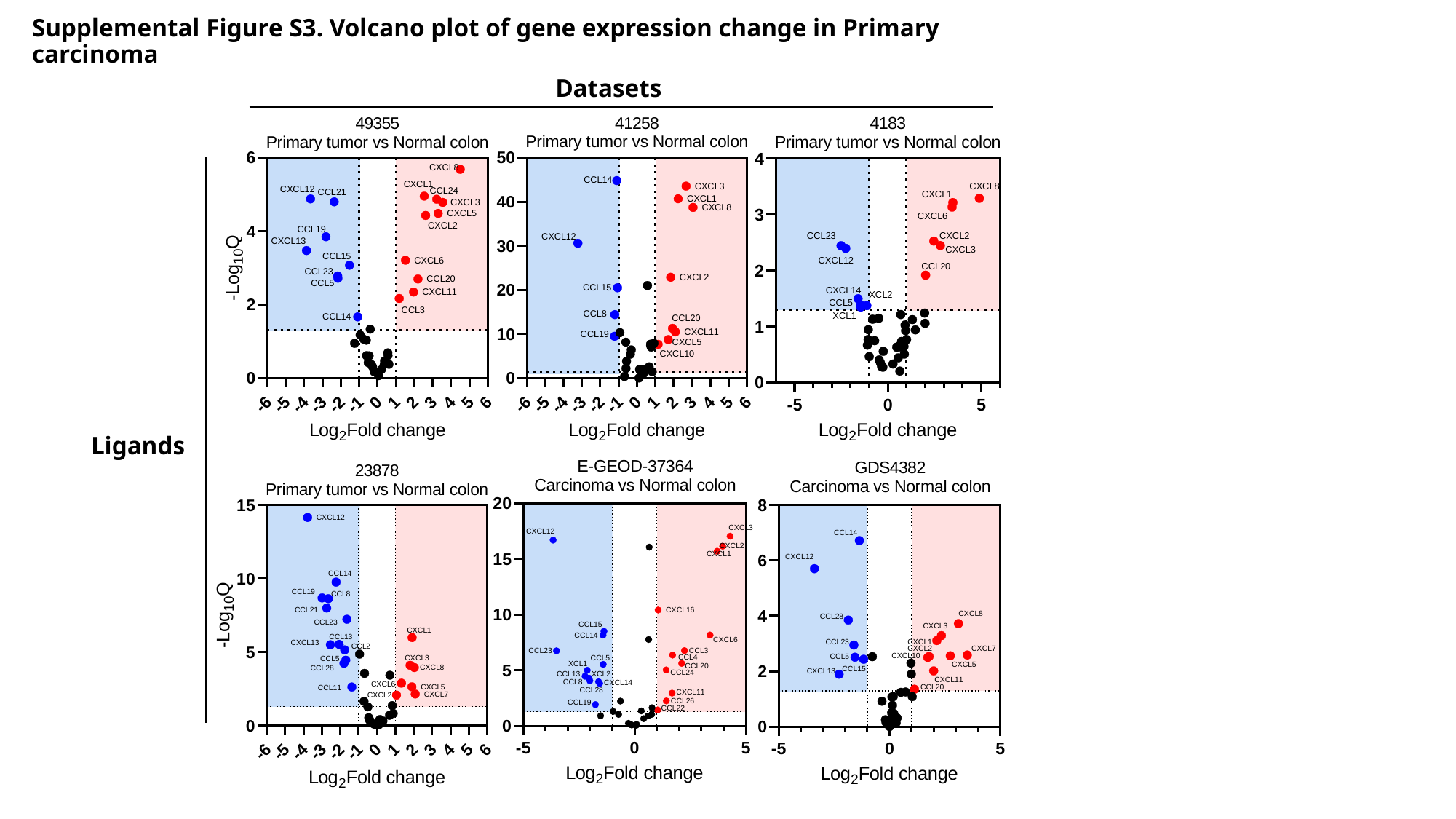

# Supplemental Figure S3. Volcano plot of gene expression change in Primary carcinoma
Datasets
Ligands

## Slide 4
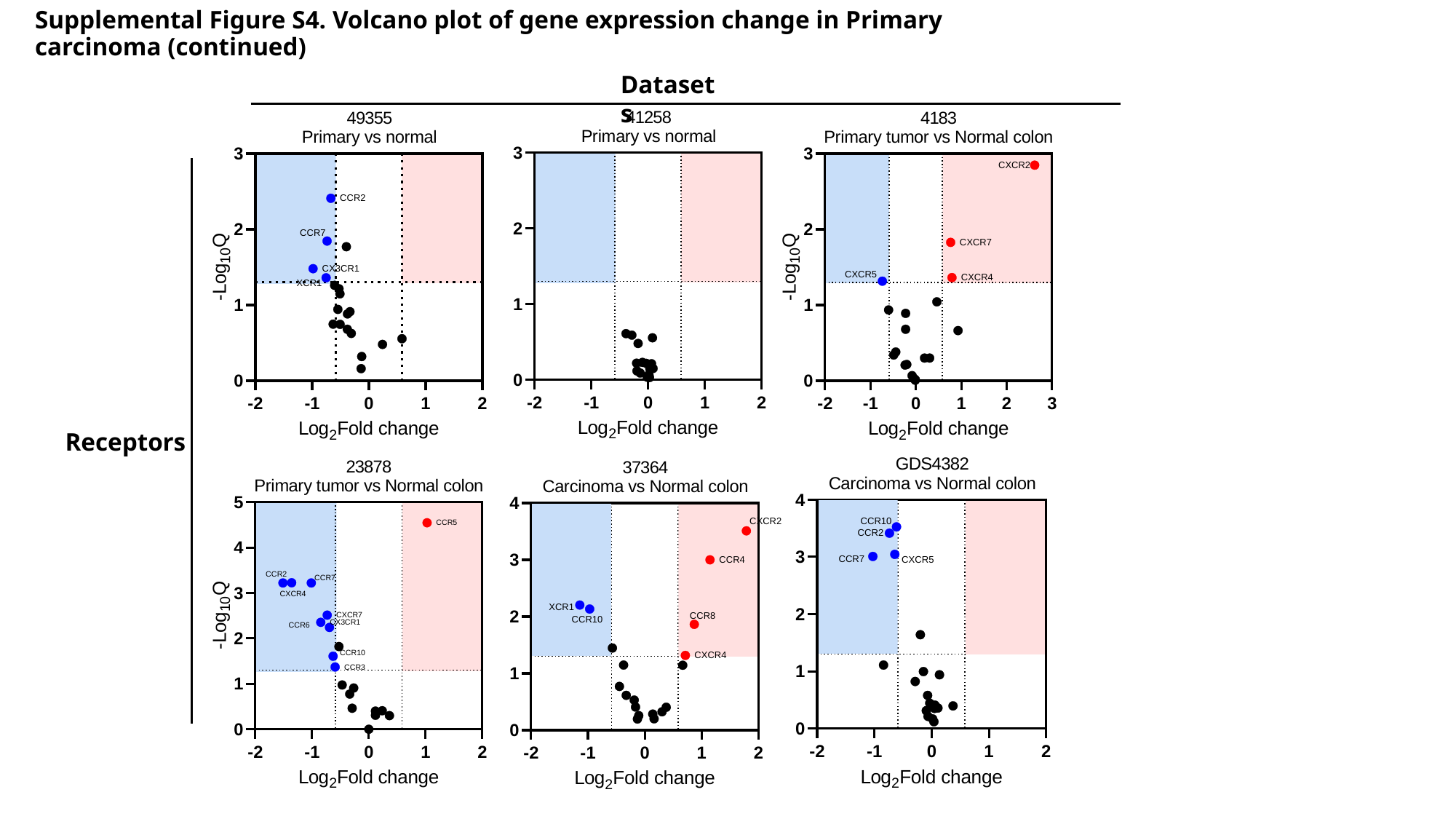

Supplemental Figure S4. Volcano plot of gene expression change in Primary carcinoma (continued)
Datasets
Receptors

## Slide 5
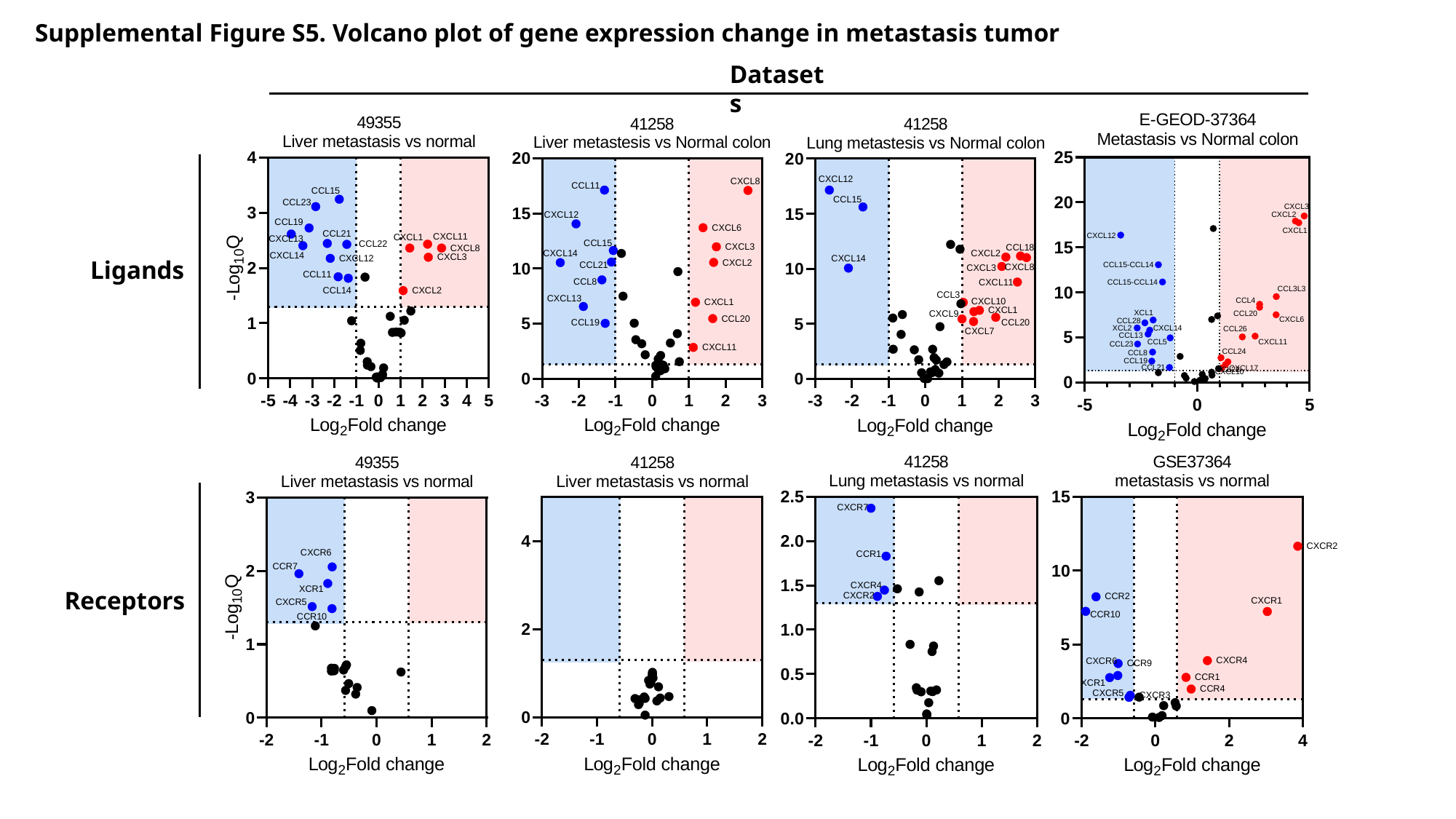

# Supplemental Figure S5. Volcano plot of gene expression change in metastasis tumor
Datasets
Ligands
Receptors

## Slide 6
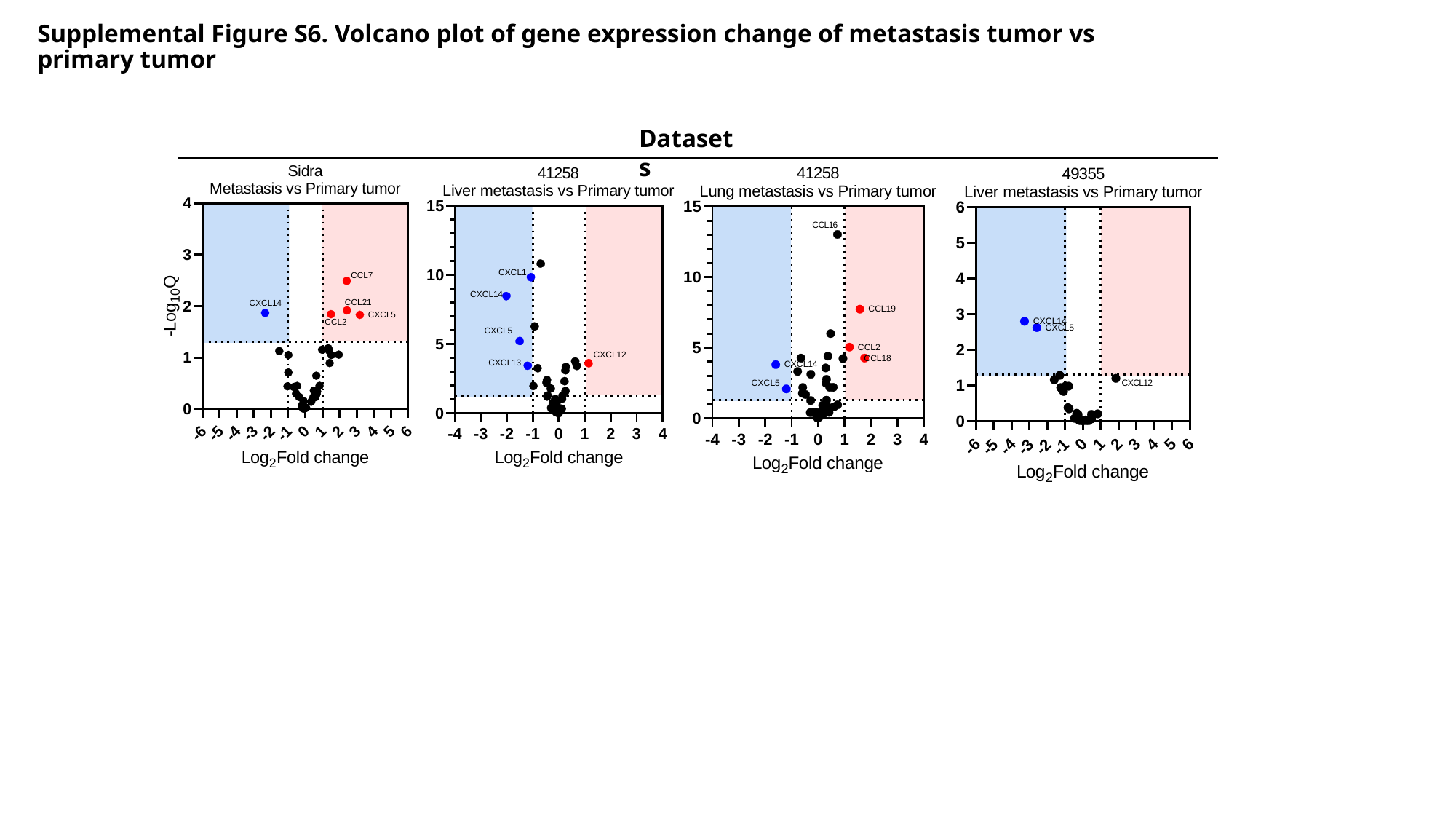

# Supplemental Figure S6. Volcano plot of gene expression change of metastasis tumor vs primary tumor
Datasets
